# Genetic Risk Factors for Longitudinal Changes in Structural MRI in Former Organolead Workers

**DOI:** 10.4061/2011/362189

**Published:** 2011-10-18

**Authors:** Bryan D. James, Brian Caffo, Walter F. Stewart, David Yousem, Christos Davatzikos, Brian S. Schwartz

**Affiliations:** ^1^Rush University Alzheimer's Disease Center, Rush University Medical Center, Room 1038, Chicago, IL 60612, USA; ^2^Department of Internal Medicine, Rush University Medical Center, Chicago, IL 60612, USA; ^3^Department of Biostatistics, Johns Hopkins Bloomberg School of Public Health, Baltimore, MD 21205, USA; ^4^Department of Epidemiology, Johns Hopkins Bloomberg School of Public Health, Baltimore, MD 21205, USA; ^5^Center for Health Research and Rural Advocacy, Geisinger Clinic, Danville, PA 17822, USA; ^6^The Russell H. Morgan Department of Radiology and Radiological Sciences, Johns Hopkins School of Medicine, Baltimore, MD 21278, USA; ^7^Department of Radiology, University of Pennsylvania School of Medicine, Philadelphia, PA 19104, USA; ^8^Department of Environmental Health Sciences, Johns Hopkins Bloomberg School of Public Health, Baltimore, MD 21205, USA; ^9^Department of Medicine, Johns Hopkins School of Medicine, Baltimore, MD 21278, USA

## Abstract

This study examined associations between polymorphisms in three genes, apolipoprotein E (*APOE*), angiotensin converting enzyme (*ACE*), and vitamin D receptor (*VDR*), and longitudinal change in brain volumes and white matter lesions (WML) as well as effect modification by cardiovascular factors and tibia lead concentrations. Two MRIs, an average of 5 years apart, were obtained for 317 former organolead workers and 45 population-based controls. Both regions-of-interest and voxel-wise analyses were conducted. *APOE ε*3/*ε*4 and *ε*4/*ε*4 genotypes were associated with less decline in white matter volumes. There was some evidence of interaction between genetic polymorphisms and cardiovascular risk factors (*ACE* and high-density lipoprotein; *VDR* and diabetes) on brain volume decline. The *VDR FokI* ff genotype was associated with an increase in WML (no association for *APOE* or *ACE*). This study expands our understanding of how genetic precursors of dementia and cardiovascular diseases are related to changes in brain structure.

## 1. Introduction

Brain volume loss [1] and increase in white matter lesions (WML) [2] are common consequences of aging, and both are related to worse cognitive function and risk of dementia [3–6]. Little is currently known about the genetic determinants of age-related brain volume loss or increase in WML or how genes may modify the effect of other environmental risk factors for these outcomes. Certain genetic polymorphisms associated with a greater risk for neurodegenerative diseases such as Alzheimer's disease (AD) in later life [7] are potential candidates. The apolipoprotein E (*APOE*) gene is the best-documented genetic risk factor for AD, with the *ε*4 allele significantly increasing the risk for AD [8–10] and cognitive decline [11]. The angiotensin converting enzyme (*ACE*) gene is associated with AD [12, 13] and WML [14]. Certain vitamin D receptor (*VDR*) gene polymorphisms have also been associated with increased risk of AD [15], cardiovascular disease, and diabetes [16, 17], and these, in turn, have been linked to WMLs [18, 19]. These genes are, therefore, strong candidates for evaluation of genetic determinants of brain volume loss and increased WML in living persons through the use of neuroimaging technology. Furthermore, because it is hypothesized that exposure to certain external agents may induce upregulation of neurodegenerative disease-associated genes [20], it is appropriate to also examine the effect of these genes in the light of gene by environment interaction. Specifically, well-known risk factors for neurodegenerative disease, including cardiovascular risk factors and occupational lead exposure, may modify the effect of certain genotypes on brain volume loss and WML.

Research has begun to evaluate relationships between genetic risk factors and structural differences in the human brain, the vast majority investigating *APOE *[21–31]. However, there are a number of limitations to this body of work, including a reliance on cross-sectional data and extrapolation of differences in structure across persons in different age ranges to within-person change. Many of these studies also involve small sample sizes, limiting power to detect differences, and some focus specifically on certain brain structures such as the hippocampus rather than structures across the whole brain. Furthermore, there has been little examination of the effect of gene by environment interaction on structural brain changes. In this paper, we report on the associations between *APOE*, *ACE*, and *VDR FokI* genetic polymorphisms and longitudinal change in brain volumes and WML from a cohort of over 350 older men who participated in two structural MRIs an average of 5 years apart. Additionally, because these genetic polymorphisms may not directly contribute to changes in brain structure, but rather may modify the effect of other risk factors (i.e., gene by environment interaction), we tested for interactions between these genes and cardiovascular risk factors as well as occupational lead exposure in determining changes in brain volumes and WML.

## 2. Methods

### 2.1. Study Design and Overview

As previously described [32–34], subjects were initially recruited during two study phases between 1994 and 2003. In phase I (1994–7), former employees of a chemical manufacturing plant in the eastern United States were identified and recruited. In phase II (2001–3), additional study participants were enrolled and the first MRI data was acquired. In phase III (2005–8), subjects who completed the first MRI were invited for a second MRI. All phases of the study were reviewed and approved by the Johns Hopkins Bloomberg School of Public Health Committee on Human Research and written informed consent was obtained from all participants.

### 2.2. Selection and Recruitment of Study Subjects

The selection, recruitment, and enrollment of former lead workers and controls (community-dwelling persons without occupational lead exposure) have been previously reported [3, 32–35]. During phase II, all participants were eligible for MRI measurement, and first MRIs were completed on 589 of 979 (60%) former lead workers and 67 of 131 (51%) controls. During phase III, a second MRI was obtained from a total of 377 persons: 317 of 589 (54%) former lead workers and 45 of 67 (67%) controls. Reasons for not obtaining a second MRI are reported elsewhere [36]. The analytic cohort herein includes the 309 former lead workers and 44 controls with two adequate MRIs (*n* = 353; 8 former lead workers and 1 control had inadequate first MRIs).

### 2.3. Data Collection

Detailed data collection methods for the first two phases of the study have been previously described [34]. The remaining description is confined to measures specifically used for the analysis presented herein. 

#### 2.3.1. Subject Interview

In phase III, the subject interview was expanded to include a number of additional study variables [37, 38]. Health outcomes (e.g., diabetes and heart disease) were ascertained by interview response to the question, “Has a doctor ever told you that you had (each condition)?” Only “yes” responses were counted; participants who answered “possible” were classified as negative for all outcomes in order to increase specificity of outcome classification. For educational attainment, information was obtained by interview on years of education, trade school, general educational development (GED) credential, and other educational certificates using previously published methods [38].

#### 2.3.2. Tibia Lead

Tibia lead, an estimate of lifetime cumulative lead dose, was available from earlier phases of the study on all former lead workers and all but one control with two MRIs. For former lead workers, current tibia lead was back-extrapolated to peak tibia lead, the estimated level at the end of employment in the factory. The measurement of tibia lead and this extrapolation to peak tibia lead are described elsewhere [33].

#### 2.3.3. Serum Tests

All serum assays were performed in the Core Laboratory of the General Clinical Research Center (Johns Hopkins Bayview Medical Center). C-reactive protein was measured by enzyme-linked immunosorbent assay (ELISA) using the American Laboratory Products Company (Salem, NH) kit, with a sample sensitivity of 0.5 ng/mL, an intrasample coefficient of variation (CV) of 6.33% and an intersample CV of 2.20%. The lipid profile was performed on a Medical Computer Systems analyzer with a sample sensitivity of 0.80%, an intrasample CV of 3.08% and an intersample CV of 3.72%. 

#### 2.3.4. Genotyping

**APOE**

Genotyping was completed using different methods in the different phases of the study as technology progressed. The method of Hixson [39] was used during phase I. In phase II, DNA was isolated using the Flexigene DNA Kit (Qiagen, Valencia, Calif, USA), and genotyping was performed using published PCR conditions [40, 41]. In phase III, DNA was isolated as for phase II. For genotyping, for determination of the C to T substitution causing the Arg112Cys and Arg158Cys polymorphisms, we performed allelic discrimination using TaqMan Probes as previously described [41] with the following modifications: (1) instead of using the nested PCR approach for the Arg112Cys polymorphism, 1X Genotyping Master Mix (Applied Biosystems, Foster City, Calif, USA) was used with 20 ng of genomic DNA and processed according to the manufacturer's recommended protocol, (2) the 1X Genotyping Master Mix was also used for the *APOE* Arg158Cys polymorphism, and (3) plate reads were performed in the 7500 Real Time PCR system to capture fluorescence, and genotypes were determined by manual clustering (Applied Biosystems 7500 software v1.2.3). Of the subjects with two MRIs, *APOE* genotyping was performed with the phase II method in 39.9% of subjects and with the phase III method in 58.5% of subjects (the rest genotyped in phase I).

**ACE**

We used a published PCR method to determine the insertion/deletion polymorphism of the *ACE* gene [42] with the following modifications: annealing time of 30 seconds and the final concentrations: 0.4 *μ*mol/L primers, 1.5 mmol/L MgCl, 200 *μ*M/L dNTPs, and 0.5 U Taq. Fragments were resolved on 2.5% agarose/TBE gels stained with EtBr. Gels were imaged and photographed with a Fuji LAS 1000 system and analyzed with Fuji Multigauge version 3.0 software.

**VDR**

Genomic DNA was isolated as for a previous study [41] from stored blood using the Flexigene DNA Kit from Qiagen (Valencia, CA). For determination of the T to C substitution causing the *VDR* 12022 polymorphism (allowing identification of FF, Ff, and ff genotypes), we performed allelic discrimination using TaqMan Probes (Applied Biosystems, Foster City, Calif, USA) using previously published methods for single nucleotide polymorphisms [41]. Allelic discrimination assays, consisting of primers and allele-specific TaqMan MGB probes labeled with 6FAM and Vic, were designed with Primer Express 2.0 and custom-ordered from Applied Biosystems (sequences of primers and probes available upon request). All reactions contained 1X assay mix, 1X TaqMan Genotyping MasterMix, and 20 ng DNA in 25 microliters. Cycling was performed in the Applied Biosystems 7500 Real Time PCR system with the following conditions: 95°C for 10 minutes and 50 cycles of amplification at 95°C for 15 seconds and 60°C for 1 minute. Following amplification, plate reads were performed as described above.

**MRI Acquisition**

For the first MRI, all subjects were imaged at the same location on the same General Electric 1.5 T Signa model as previously described [34]. For the second MRI, a 3 T General Electric scanner was utilized. T1-weighted images were acquired using an SPGR sequence (TE = 8 ms, TR = 21 ms, flip angle = 30°, FOV = 24 cm). Axial PD/T2 (TR/TE/TE2 = 2,200/27/120) and FLAIR (TR/TE/T1 = 8,000/100/2000) images were also acquired for WML grading.

**Clinical MRI Review and Assignment of WML Grade Scores**

MRIs were reviewed to exclude urgent or emergent brain disease and subjects and their physicians were notified if present [43]. MRIs were assigned a WML grade score by a trained neuroradiologist using the Cardiovascular Health Study (CHS) ten-point (0 to 9) scale [44, 45], as previously reported [34], which allowed for analysis of change in ratings.

### 2.4. Image Analysis

The methods to obtain regional and voxel-wise volumes, including skull stripping, segmentation, registration, and transformation to regional analysis of volumes examined in normalized space (RAVENS), were completed using published methods [34, 36, 46–50]. Due to changes in scanner technology and pulse sequences, we employed specialized image analysis methods that minimized the discontinuity between the two scans. We used the CLASSIC algorithm [51], which employs a 4-dimensional segmentation framework in which the baseline and follow-up scans are considered jointly to minimize discrepancies between the two segmentations and better estimate longitudinal change. This algorithm has been previously validated [51].

### 2.5. Statistical Analysis

The purpose of the present analysis was to first determine if genotypes for three different candidate genes were associated with changes in brain volumes and WML and then evaluate whether the genes modified relations of cardiovascular factors and tibia lead with changes in brain volumes and WML. Multiple linear regression was used to evaluate associations of the polymorphisms with change in brain volumes using both ROI-based and voxel-wise approaches as well as change in CHS scores (WML). All regression models were adjusted for baseline age, duration of time between MRIs, control status (i.e., former lead worker versus control), height (cm), and education [38] and baseline ROI volume for ROI analysis or baseline CHS score for the WML analysis. Results were similar for models that did not include terms for baseline ROI volume or CHS score (not presented). Cross-product terms were used to evaluate effect modification.

Because lead is associated with smaller brain volumes [34], we first evaluated whether the association between genotypes and change in brain volumes or WML differed between former lead workers and population-based controls or, within former lead workers, the associations of genotypes with MRI outcomes differed by peak tibia lead (PTL) level. There was no evidence that associations of interest differed by control status, so we proceeded with our main analyses using data from both lead workers and controls. Furthermore, a separate analysis found no association between PTL and change in brain volumes [36]. We incorporated the results of PTL by gene interactions in former lead workers into our analyses as described below.

#### 2.5.1. ROI-Based Approach

We modeled change in 20 previously selected ROI volumes consistent with our prior published reports (as listed in Table 2) [34]. For bilateral structures, the volume represented the sum of right and left structures to minimize multiplicity concerns, but analyses were also performed separately for change in left- and right-sided ROI volumes (data not reported). We did not formally adjust for multiple comparisons in the analysis, choosing instead to report unadjusted *P* values and the number of regressions. 

We first examined the relationship between each genotype and change in ROI volumes (core models). We then separately examined the relationships between cardiovascular risk factors (hypertension (HTN; yes versus no), cardiovascular disease (CVD; yes versus no), diabetes mellitus (DM; yes versus no), total cholesterol, high-density lipoprotein (HDL), low-density lipoprotein (LDL), and C-reactive protein (CRP)) and change in brain volumes. To evaluate effect modification by genotypes on relations of cardiovascular risk factors with change in volumes or WMLs, we added a term for cardiovascular risk factors (separately) and a cross-product term for genotype ∗ risk factor to the core models. Effect modification by genotypes on age relations were also examined in separate models with a cross-product term for genotype ∗ age at baseline. We then examined effect modification by genotype on the relationship of CHS score at baseline, as well as change in CHS score across visits, with change in ROI volumes. Finally, in former lead workers, we examined interactions of PTL and genotypes on change in ROI volumes. Model diagnostics were used to evaluate influence and normality. 

#### 2.5.2. Voxel-Wise Approach

The relationship between genotypes and change in voxel volumes was modeled controlling for the aforementioned covariates using multivariate permutation testing in the R statistical programming language (http://www.cran.r-project.org/). The SPM5 package (Statistical Parametric Software, Functional Imaging Laboratory, Wellcome Department of Imaging Neuroscience, University College London, 2003) was used to perform smoothing using a 3D isotropic Gaussian filter and MRIcro [52] to display results. Statistical significance was evaluated using a permutation approach that controlled for confounding variables. The maximum cluster size and cluster peak above the threshold was used to define a conservative permutation distribution on cluster sizes and peaks that, when compared to the observed cluster sizes and peaks, controls for multiple comparisons.

#### 2.5.3. White Matter Lesions

Linear regression was used to model change in WML grade scores controlling for covariates and evaluating the same effect modification variables. As in the ROI-based analysis, we examined the relationships between cardiovascular risk factors and change in WML, and then effect modification by genotype on relationships of cardiovascular risk factors with change in WML. Effect modification by genotype on the relationship of age and change in WML was also examined. In former lead workers, we examined interactions of PTL and genotypes on change in WML. 

## 3. Results

### 3.1. Descriptive Summary of Study Subjects

Basic descriptive characteristics of the 353 subjects with two valid MRIs are presented elsewhere [36]. In short, the mean (SD) age was 65.1 (7.9) years (range: 48–82), 93% had a high school education, and 90% were white. Seven subjects were missing *ACE* genotyping, 6 subjects were missing *ACE* and *VDR* genotyping, and 1 subject was missing data for *ACE*, *VDR*, and *APOE*. With the exception of CRP by *APOE* genotype and diabetes by *VDR* genotype, there were no differences in distributions of cardiovascular risk factors by genotype (Table 1). There were no differences in *APOE*, *ACE*, or *VDR* genotypes by control status (data not shown). There were no differences in *APOE* or *VDR* genotypes by MRI status (i.e., zero versus one versus two MRIs; data not shown); we did not perform *ACE* genotyping on persons without two MRIs. Controls had significantly lower mean (SD) levels of total cholesterol (182.0 (31.5) versus 200.9 (40.8), *P* = 0.004) and LDL (97.5 (29.4) versus 114.5 (34.8), *P* = 0.003), and higher levels of CRP (3.3 (3.8) versus 2.4 (2.5), *P* = 0.04) than former lead workers.

### 3.2. Change in ROI Volumes

As presented elsewhere in more detail [36], the volumes of all ROIs except for occipital WM declined from the first to the second MRI over an mean (SD) time of 5.0 (0.4) years, with a more substantial decline in gray (−24.4 cm^3^) versus white (−5.4 cm^3^) matter. On average, total brain volume declined an average of 30 cm^3^.

**Cardiovascular Risk Factors and WML**

There was little consistent evidence of a main effect of cardiovascular risk factors on change in ROI volumes. However, higher HDL was associated with more decline in 5 ROI volumes: total brain volume (*β*(SE) = −0.183(0.078), *P* = 0.02), total WM (*β*(SE) = −0.101 (0.047), *P* = 0.03), parietal WM (*β*(SE) = −0.038 (0.011), *P* < 0.001), cingulate gyrus (*β*(SE) = −0.009 (0.004), *P* = 0.01), and hippocampus (*β*(SE) = −0.004 (0.001), *P* = 0.006). More WML at baseline and change in WML were not associated with change in ROI volume.

**APOE**

**Figure 1:**
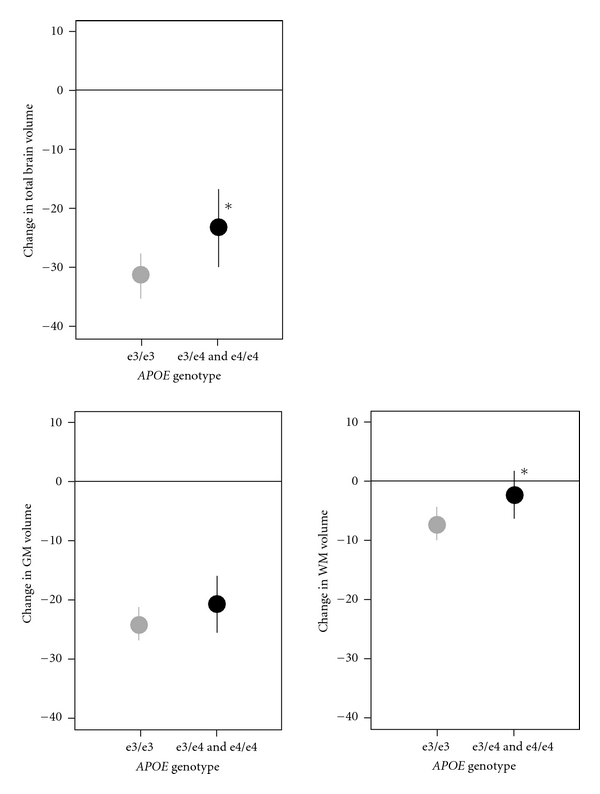
Change in total brain, GM, and WM volumes by *APOE* genotype. The grey and black lines are estimated change in volumes (mean ± 95% confidence interval) for the *APOE ε*3/*ε*3 and *APOE ε*3/*ε*4 + *ε*4/*ε*4 groups, respectively. The asterisk indicates that the estimated change for the *ε*3/*ε*4 + *ε*4/*ε*4 group is significantly different than for the *ε*3/*ε*3 group (*P* < 0.05).

There were consistent associations of *APOE* genotype with change in ROI volumes (Table 2). Results are only presented for the *ε*3/*ε*4 and *ε*4/*ε*4 genotypes combined, from a model that also included terms for *ε*2/*ε*2 plus *ε*2/*ε*3 and *ε*2/*ε*4 (with *ε*3/*ε*3 as the reference group). The positive beta coefficients indicate less decline for those with the *ε*3/*ε*4 or *ε*4/*ε*4 genotypes (e.g., the TBV for persons with the *ε*3/*ε*4 or *ε*4/*ε*4 genotypes declined an average of 23 cm^3^ versus 31 cm^3^ for persons with the *ε*3/*ε*3 genotype (Figure 1)). The differences in ROI volume declines by *APOE* genotype were largest and most consistent for changes in white matter volumes.

**Figure 2:**
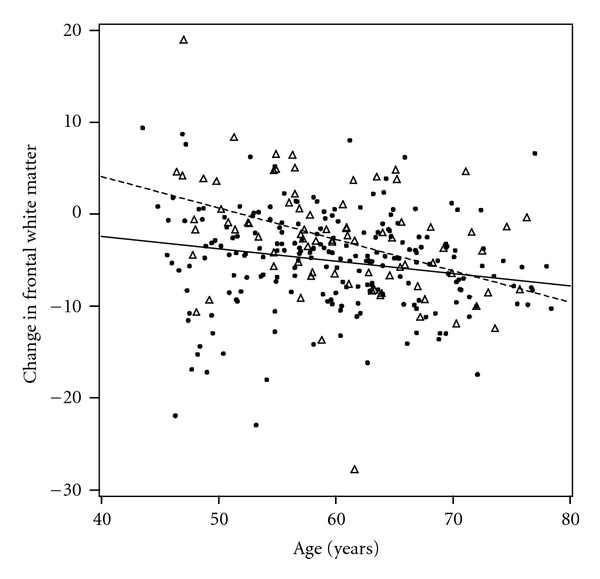
Effect modification by *APOE* genotype on relation of age with change in frontal WM volume for *APOE ε*3/*ε*3 (black dots, solid regression line) and *ε*3/*ε*4 plus *ε*4/*ε*4 groups (triangles, dashed regression line). The slopes of the two lines were different (*P* < 0.05).

There was evidence that *APOE* genotype modified relations of age with change in ROI volumes. Persons who were older at baseline and had the *ε*3/*ε*4 or *ε*4/*ε*4 genotype experienced more decline in the following ROI volumes: frontal WM (*β*(SE) = −0.207 (0.086), *P* = 0.02), parietal WM (*β*(SE) = −0.115 (0.048), *P* = 0.02), corpus callosum (*β*(SE) = −0.023 (0.007), *P* = 0.001), hippocampus (*β*(SE) = −0.013 (0.006), *P* = 0.02), and amygdala (*β*(SE) = −0.008 (0.003), *P* = 0.02) (frontal WM displayed in Figure 2. There were no consistent interactions between cardiovascular risk factors and genotype for change in any ROI volume. 

**Figure 3:**
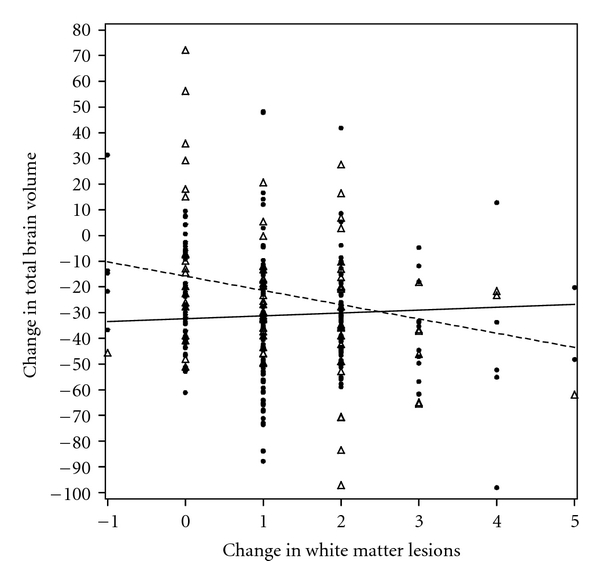
Effect modification by *APOE* genotype on relation of change in WML and change in total brain volume for *APOE ε*3/*ε*3 (black dots, solid regression line) and *ε*3/*ε*4 plus *ε*4/*ε*4 groups (triangles, dashed regression line). The slopes of the two lines were different (*P* < 0.05).

We next evaluated whether relations among change in WML and change in ROI volumes were modified by *APOE* genotype. In models that included terms for change in WML and a cross-product for *APOE* genotype ∗ change in WML, there was evidence of such effect modification. Persons who had *APOE ε*3/*ε*4 or *ε*4/*ε*4 genotype and an increase in WML experienced less decline in the following ROI volumes; total brain volume (*β*(SE) = −6.658 (2.402), *P* = 0.006), total WM (*β*(SE) = −3.727 (1.419), *P* = 0.009), frontal WM (*β*(SE) = −1.717 (0.611), *P* = 0.005), temporal WM (*β*(SE) = −0.806 (0.358), *P* = 0.03), parietal WM (*β*(SE) = −1.183 (0.341), *P* = 0.001), medial structures (*β*(SE) = −0.802 (0.304), *P* = 0.009), cingulate gyrus (*β*(SE) = −0.251 (0.116), *P* = 0.03), insula (*β*(SE) = −0.242 (0.081), *P* = 0.003), corpus callosum (*β*(SE) = −0.117 (0.052), *P* = 0.03), internal capsulate (*β*(SE) = −0.125 (0.048), *P* = 0.01), and hippocampus (*β*(SE) = −0.139 (0.040), *P* = 0.001) (see, e.g., Figure 3).

**ACE**

There were no associations between *ACE* genotype and change in ROI volumes. There was no evidence that *ACE* genotype modified relations of age with change in ROI volumes. There was evidence that *ACE* genotype modified relations of HDL with change in ROI volumes. Persons with greater HDL who had the I/I genotype experienced less decline in the following ROIs: total brain volume (*β*(SE) = 0.533 (0.211), *P* = 0.01), total WM (*β*(SE) = 0.342 (0.127), *P* = 0.007), frontal WM (*β*(SE) = 0.131 (0.055), *P* = 0.02), parietal WM (*β*(SE) = 0.080 (0.030), *P* = 0.009), occipital WM (*β*(SE) = 0.034 (0.017), *P* = 0.04), and hippocampus (*β*(SE) = 0.007 (0.004), *P* = 0.04). There was no evidence that *ACE* genotype modified relations of any other cardiovascular risk factors or WML scores with change in ROI volumes.

**VDR**

There were no associations between *VDR* genotype and change in ROI volumes. There was no evidence that *VDR* genotype modified relations of age with change in ROI volumes. There was evidence that *VDR* genotype modified relations of diabetes with change in ROI volumes. Persons who had diabetes and were heterozygous for the *VDR FokI* Ff genotype experienced less decline in the following ROIs: total WM (*β*(SE) = 9.873 (4.102), *P* = 0.02), frontal WM (*β*(SE) = 3.557 (1.755), *P* = 0.04), temporal WM (*β*(SE) = 2.412 (1.013), *P* = 0.02), occipital WM (*β*(SE) = 1.281 (0.537), *P* = 0.02), internal capsule (*β*(SE) = 0.336 (0.137), *P* = 0.02), and entorhinal cortex (*β*(SE) = 0.153 (0.062), *P* = 0.01). There was no consistent evidence that *VDR* genotype modified relations of any other cardiovascular risk factors with change in ROI volumes. There was evidence that *VDR* modified relations of change in WML scores and change in ROI volumes. Persons who were homozygous for the *VDR FokI* ff genotype and had an increase in WML experienced more decline in the following ROI volumes: total GM (*β*(SE) = −4,570 (2.089), *P* = 0.03), frontal GM (*β*(SE) = −1.402 (0.604), *P* = 0.02), and parietal GM (*β*(SE) = −0.696 (0.319), *P* = 0.03), but *less *decline in two ROIs: frontal WM (*β*(SE) = 1.513 (0.723), *P* = 0.04), and parietal WM (*β*(SE) = 0.895 (0.401), *P* = 0.03).

**Lead By Gene Interaction**

In former lead workers, there was little evidence that PTL modified the relationship of candidate genes with change in ROI volumes. A significant interaction of PTL with *APOE ε*3/*ε*4 or *ε*4/*ε*4 genotype was found in only one ROI, occipital GM (*β*(SE) = 0.031 (0.016), *P* = 0.045).

**Change in Voxel Volumes**

**Figure 4:**
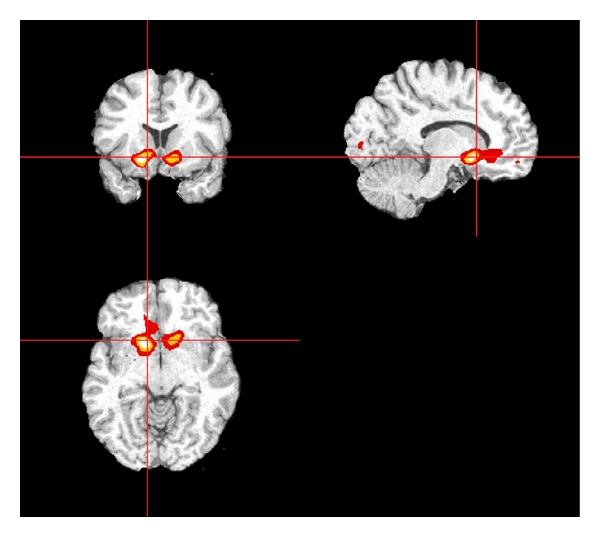
Largest significant clusters where *APOE ε*4/*ε*4 or *ε*3/*ε*4 genotypes were associated with less decline in WM volume for statistical maps based on family-wise error rate corrected *P* value thresholding from permutation testing. Results are shown for relevant coronal (upper left), sagital (upper right) and axial (lower) slices. Statistical significance was based on suprathreshold cluster-level permutation testing. Colors represent voxels satisfying *P* value thresholds of 0.001, 0.0001, and 0.00001, respectively.

In a parallel analysis, results were substantively similar using a voxel-wise approach. The supra-threshold clusters for the association of *ACE* genotype and *VDR* genotype with change in GM and WM volume were within the range expected by chance (not shown). The adjusted association between *APOE ε*3/*ε*4 or *ε*4/*ε*4 genotype and change in voxel volumes identified large supra-threshold clusters in WM, whose sizes were above the distribution of the maximum cluster size under the null hypothesis (largest association cluster depicted in Figure 4).

### 3.3. Change in WML Grade Score

**Figure 5:**
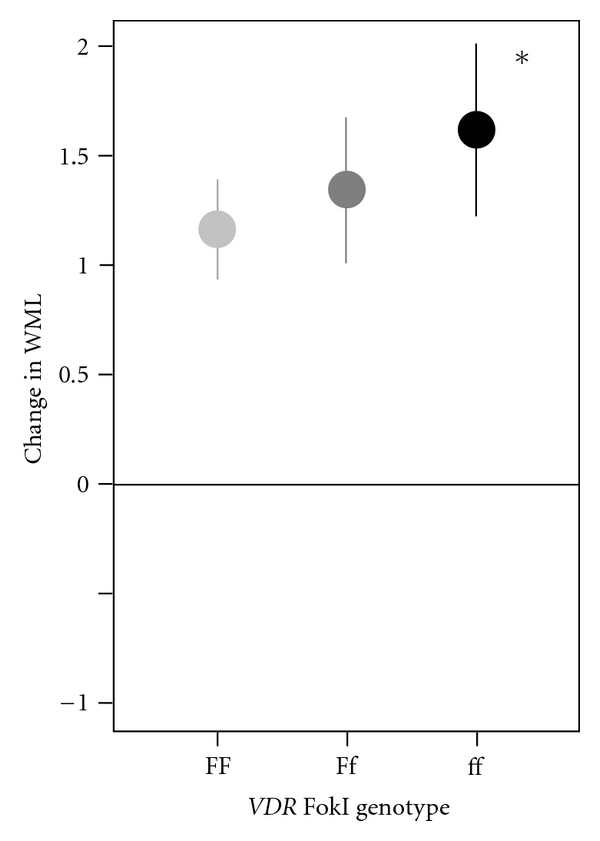
Change in WML grade score by *VDR FokI* genotype. The light gray, dark gray, and black lines are for groups with the FF, Ff, and ff genotypes, respectively. The asterisk indicates that the estimated change in WML scores for genotype was significantly different from the FF genotype.

Summary statistics for change in CHS WML scores between the first and second MRI have been previously reported [36]. In brief, 74% of the sample showed increased WML over followup. The *APOE* and *ACE* genotypes were not associated with changes in WML scores. In adjusted analysis, controlling for age, duration between MRIs, control status, height, education, and baseline CHS score, the *VDR FokI* polymorphism was associated with increases in WML in a gene-dose-dependent fashion, with beta coefficients (SE, *P*-value) of 0.18 (0.12, *P* = 0.13) and 0.45 (0.16, *P* = 0.006) for Ff and ff genotypes, respectively. This indicates, for example, that, on average, subjects with the ff genotype had CHS scores that increased 0.45 categories higher than did those with the FF genotype (Figure 5). These associations did not change when baseline CHS score was removed from the model. In former lead workers, there was no evidence of interactions of genes with PTL.

## 4. Discussion

In this cohort of nondemented older men with two MRI scans an average of five years apart, we examined relations of three genetic polymorphisms with longitudinal change in brain volumes and WMLs, Our main findings were that the *APOE ε*3/*ε*4 and *ε*4/*ε*4 genotypes were associated with less decline in brain volumes over time, especially in WM, and that these genotypes modified the relationship of age as well as change in WML with change in brain volumes. We also found that the *VDR FokI* ff genotype was associated with an increase in WMLs. There was some evidence that the genotypes modified relations of cardiovascular risk factors with change in both ROI volumes and WMLs, but these findings were not consistent across brain regions or consistent across risk factors. There was no evidence that genotypes modified relations of lead levels with change in ROI volumes and WMLs in former lead workers. These findings give us some insight into the genetic determinants of structural changes in the brain that may contribute to cognitive impairments in later life.

A number of studies have examined the relation between *APOE* genotype and brain structure. The *ε*4 allele has been associated with smaller total brain, gray matter, hippocampus, amygdala, and corpus callosum volumes, and more WMLs [21–24]. However, there are a number of studies that have found no association between *APOE* and brain volumes [25, 26] or WML [26] in healthy samples, and at least three studies in AD patients have found an association between *ε*4 and *larger* volumes [27–29]. The majority of these studies used cross-sectional study designs in which change in structure volumes across age ranges is extrapolated from inter-individual differences in age. Very little research has focused on how these genetic risk factors relate to longitudinal intraindividual changes in brain structure. In nondemented cohorts, one longitudinal study found an association of the *ε*4 allele with greater hippocampus volume loss [30], while another found a nonsignificant trend for a relation between the *ε*4 allele and greater brain atrophy [31]. One longitudinal study on APOE genotype and WMLs has been conducted, which found an increase in WMLs in *ε*4/*ε*4 individuals only [53]. There has been little published research on *ACE* polymorphisms and differences in brain structure. One study found an association between the I/I genotype and smaller hippocampus and amygdala volumes in women only, but no association with WML [54] and another found no relation to volume or WML [55]. A recent review found evidence of association between *ACE* I/D polymorphism and WML from 9 cross-sectional studies, but cautioned against publication bias [56]. We were unable to identify any prior studies of *VDR* genotype and brain structure.

Our results are not consistent with the small number of studies that have previously examined the relations between the *APOE ε*4 allele and change in brain volumes in nondemented cohorts. We found that subjects with the *ε*4 allele had less rather than more decline in volumes of brain structures compared to those without the allele. This finding is unexpected in light of the established relation between the *ε*4 allele and neurodegenerative diseases such as AD and the concomitant brain atrophy experienced by individuals who have these diseases. However, given the paucity of evidence regarding this relationship, these findings should be treated as preliminary and may suggest a different pathway from gene to AD expression as mediated through structural changes in the brain than have been previously recognized. For example, the association between *ε*4 and less decline in volume was strongest in WM; this could be consistent with an adverse effect in persons with the *ε*4 allele if the slower rate of WM volume loss is due to inflammation, edema, swelling of cells, or other changes in WM that are present in early lesions in these relatively young study subjects [57]. This hypothesis may be supported by the accompanying finding that higher levels HDL, usually considered protective against vascular events, was associated with more WM decline as well. Alternatively, these findings may align with the emerging theory of *APOE* antagonistic pleiotropy in which the *ε*4 allele confers an advantage at younger ages while producing detrimental neurocognitive consequences in later life [58]. This is supported by our finding of an interaction between age and *ε*4 status on WM decline in which persons with *ε*3/*ε*4 or *ε*4/*ε*4 genotype experience less decline than *ε*3/*ε*3 carriers at earlier ages, but this difference attenuates and actually reverses after the age of 70, after which *ε*3/*ε*4 or *ε*4/*ε*4 experience more decline. Further, there is evidence that *APOE* antagonistic pleiotropy is related to integrity of the cholinergic system [59]; the most robust associations between *ε*3/*ε*4 or *ε*4/*ε*4 genotype and less decline was found in the basal forebrain (Figure 4), a region considered to be the major cholinergic output of the brain. 

We also observed effect modification by *APOE* genotype on the relation of change in WML with change in brain volumes. Persons with *ε*3/*ε*4 or *ε*4/*ε*4 genotypes experience *more *decline in brain volumes with increases in WML. Notably, cardiovascular risk factors were not associated with increased WML, perhaps indicating that both decline in WM and progression of WML may not have linear relationships with traditional risk factors for cognitive impairment over the life course. Finally, our study is the first to report on an association between *VDR FokI* genotype and change in WML. This finding needs to be replicated before any conclusions can be drawn, but connections between *VDR*, cardiovascular disease, and WMLs gives this finding biological plausibility [16, 19].

The study had several strengths, including larger sample size than most prior studies, longitudinal design, use of ROI-based and voxel-wise analyses, relatively long duration between scans, and analysis of WMLs as determined by application of the CHS WML grading method. The main strength of this study was the ability to examine intraindividual change in brain structures over a 5-year period using longitudinal data. This provides a more valid measure of change and predictors of change than extrapolating an estimate of change from separate individuals across a range of ages using cross-sectional data. 

A limitation of this study is the selected nature of the cohort, which was made up entirely of men, most of whom had histories of occupational lead exposure. However, general population samples have shown tibia lead levels similar to this cohort [60, 61], consistent with documentation that all Americans over the age of 50 years had significant environmental lead exposure [62]. Thus, our ability to adjust for and examine interactions with lead is also a strength of this study. The fact that prior studies of older Americans have not considered this ubiquitous neurotoxicant that influences brain volumes [34] could be an important source of confounding. The ubiquity of lead exposure could also mask a potential gene by lead interaction, resulting in a gene appearing to exert a main effect [63]. However, a gene by lead interaction was not observed for change in brain volumes or increase in WML.

An important consideration that could affect the internal validity of these results is selection bias, as persons who had an MRI scan may not be representative of the total cohort. In previous papers, we reported that there was unlikely to be meaningful selection bias, and if present, would likely mask rather than spuriously create associations [34, 36]. A methodological challenge was changes in the scanner technology between the two MRI scans. We attempted to minimize the problems introduced by these changes by using an image analysis technique that was specifically developed and validated for longitudinal studies that is more likely to underestimate rather than overestimate longitudinal brain changes [51].

In conclusion, this analysis adds to the emerging body of literature on genetic contributions to brain changes in later life. The findings suggest that early WM lesions in middle-aged persons with the *APOE* *ε*4 allele may initially be space-occupying, due to inflammation, edema, or swelling of cells, but that with advancing age and increases in WM lesions, persons with the *ε*4 allele experience more volume loss. This analysis is also one of the first to show an association between *VDR *genotype and changes in WM lesions. 

## Figures and Tables

**Table 1:** Distribution of cardiovascular risk factors by genotype.

Genotype	*N* (%)	HTN *N* (%)	CVD *N* (%)	Diabetes *N* (%)	Total cholesterol mean (SD)	HDL mean (SD)	LDL mean (SD)	CRP mean (SD)
*APOE* (*n* = 352)^1^								
*ε*2/*ε*3 + *ε*2/*ε*2	46 (14)	20 (43)	11 (24)	10 (22)	196.7 (49.4)	52.7 (15.6)	107.0 (41.9)	2.7 (2.6)
*ε*3/*ε*3	214 (61)	116 (54)	23 (11)	39 (18)	198.8 (39.7)	50.2 (13.9)	111.8 (34.4)	2.7 (2.9)
*ε*2/*ε*4	11 (3)	6 (55)	2 (18)	0 (0)	196.9 (39.8)	44.3 (14.8)	121.3 (27.6)	2.5 (2.6)
*ε*3/*ε*4 + *ε*4/*ε*4	81 (23)	42 (52)	9 (11)	15 (19)	199.2 (36.6)	48.3 (12.9)	116.2 (31.2)	1.7 (1.8)
		*P* = 0.62	*P* = 0.094	*P* = 0.42	*P* = 0.99	*P* = 0.19	*P* = 0.43	*P* = 0.02

*ACE* (*n* = 339)^2^								
I/I	64 (19)	38 (59)	8 (13)	10 (16)	200.2 (38.9)	52.5 (14.9)	111.9 (34.7)	2.1 (1.8)
I/D	137 (40)	67 (49)	12 (9)	26 (19)	196.7 (39.0)	49.8 (13.3)	112.0 (31.8)	2.4 (2.6)
D/D	138 (41)	74 (54)	22 (16)	25 (18)	198.9 (42.6)	48.7 (14.4)	112.5 (37.7)	2.7 (2.9)
		*P* = 0.37	*P* = 0.20	*P* = 0.85	*P* = 0.82	*P* = 0.20	*P* = 0.99	*P* = 0.32

*VDR* (*n* = 346)								
FF	125 (36)	60 (48)	17 (14)	15 (12)	198.6 (39.1)	49.4 (14.8)	111.9 (33.3)	2.4 (2.7)
Ff	168 (49)	90 (54)	19 (11)	34 (20)	199.1 (40.9)	49.5 (13.7)	114.0 (36.0)	2.6 (2.7)
ff	53 (15)	31 (58)	8 (15)	14 (26)	197.3 (42.6)	52.2 (13.1)	109.3 (34.4)	1.9 (2.2)
		*P* = 0.40	*P* = 0.72	*P* = 0.047	*P* = 0.96	*P* = 0.43	*P* = 0.68	*P* = 0.21

^1^Certain genotypes were combined for analysis, resulting in following analytic groups: (1) *ε*3/*ε*4 and *ε*4/*ε*4, (2) *ε*2/*ε*2 and *ε*2/*ε*3, (3) *ε*2/*ε*4, (4) *ε*3/*ε*3 (reference group). ^2^I: insertion, D: deletion.

**Table 2:** Regression^a^ results for delta ROI models for former lead workers and controls (*N* = 352), adjusting for confounding variables.

	*APOE*	*ACE*		*VDR*	
	*ε*3/*ε*4 + *ε*4/*ε*4^c^	I/D, I/I^d^		Ff, ff^e^	
ROI^b^	Beta (SE)	Beta (SE)		Beta (SE)	
TBV	8.045 (2.621)***	4.498 (2.477)	*−*2.709 (3.134)	2.188 (2.429)	0.537 (3.373)

TOTAL GM	3.320 (1.975)*	2.135 (1.839)	*−*1.236 (2.328)	1.733 (1.806)	*−*0.038 (2.508)
FRONT GM	1.076 (0.575)*	0.645 (0.534)	*−*0.591 (0.677)	0.585 (0.524)	0.288 (0.729)
OCCIP GM	0.241 (0.213)	0.187 (0.198)	*−*0.083 (0.251)	0.254 (0.194)	0.106 (0.271)
PARI GM	0.612 (0.301)**	0.636 (0.282)**	*−*0.041 (0.357)	0.104 (0.276)	*−*0.011 (0.383)
TEMP GM	0.619 (0.485)	0.677 (0.457)	0.046 (0.578)	0.709 (0.443)	*−*0.063 (0.616)

TOTAL WM	5.059 (1.554)***	2.402 (1.491)	*−*1.398 (1.887)	0.548 (1.451)	0.309 (2.012)
FRONT WM	2.331 (0.666)***	0.839 (0.639)	*−*0.825 (0.309)	*−*0.080 (0.623)	*−*0.248 (0.864)
OCCIP WM	0.818 (0.203)***	0.176 (0.195)	*−*0.051 (0.246)	0.140 (0.190)	0.052 (0.263)
PARI WM	0.921 (0.374)**	0.378 (0.358)	*−*0.291 (0.452)	0.071 (0.347)	0.111 (0.481)
TEMP WM	0.557 (0.389)	0.242 (0.367)	*−*0.477 (0.464)	*−*0.148 (0.358)	0.130 (0.495)

ERC	0.032 (0.024)	0.008 (0.023)	0.016 (0.029)	*−*0.001 (0.022)	*−*0.013 (0.031)
AMYG	0.035 (0.024)	0.033 (0.023)	*−*0.043 (0.029)	0.013 (0.022)	*−*0.039 (0.031)
HIPPO	0.070 (0.044)	0.044 (0.042)	*−*0.020 (0.053)	0.050 (0.040)	0.057 (0.056)
CEREB	0.102 (0.473)	0.158 (0.447)	*−*0.043 (0.568)	0.650 (0.432)	0.447 (0.598)
MEDIAL	0.898 (0.332)***	0.231 (0.315)	*−*0.361 (0.396)	0.207 (0.306)	0.044 (0.424)
INSULA	0.128 (0.088)	0.092 (0.083)	*−*0.146 (0.104)	0.010 (0.081)	*−*0.051 (0.113)
CINGULATE	0.236 (0.124)*	0.149 (0.116)	*−*0.047 (0.147)	0.050 (0.114)	*−*0.107 (0.159)
CORP CALL	0.055 (0.057)	0.005 (0.054)	0.039 (0.068)	*−*0.029 (0.052)	*−*0.033 (0.072)
INT CAPS	0.151 (0.052)***	−0.002 (0.050)	*−*0.059 (0.064)	*−*0.028 (0.049)	*−*0.058 (0.068)

*0.05 < *P* < 0.10; **0.01 < *P* < 0.05; ***P < 0.01.

^a^Models adjusted for height, baseline ROI, control status, duration between MRIs, and education.

^b^ROI: region of interest; TBV: total brain volume (TBV1 = TBV at first MRI); GM: gray matter; FRONT: frontal; OCCIP: occipital; PARI: parietal; TEMP: temporal; WM: white matter; ERC: entorhinal cortex; AMYG: amygdala; HIPPO: hippocampus; CEREB: cerebellum; MEDIAL: medial structures (bilateral amygdala, cuneus, entorhinal cortex, hippocampal formation, lingual gyrus, medial front-orbital gyrus, medial frontal gyrus, medial occipitotemporal gyrus, parahippocampal gyrus, perirhinal cortex, precuneus, and uncus); CORP CALL: corpus callosum; INT CAPS: internal capsule.

^c^Compared to *APOE*3-3 as reference group; model also included terms for 22 + 23 and 24.

^d^Compared to D/D (homozygous for deletion) as reference group.

^e^Compared to FF as reference group.
